# Recent advances in drug repurposing for cancer immunomodulation emerging strategies, mechanistic insights, and clinical translation

**DOI:** 10.3389/fonc.2026.1763769

**Published:** 2026-03-20

**Authors:** Jonaid Ahmad Malik, Hend Talkhan, Farhat Fatima, Umme Hani

**Affiliations:** 1 Department of Biomedical Engineering, Indian Institute of Technology, Ropar, India; 2 Clinical Pharmacy Department, College of Pharmacy, King Khalid University, Abha, Saudi Arabia; 3 Department of Pharmaceutics, College of Pharmacy, Prince Sattam Bin Abdulaziz University, Al Kharj, Saudi Arabia; 4 Department of Pharmaceutics, College of Pharmacy, King Khalid University, Abha, Saudi Arabia

**Keywords:** cancer, drug repurposing, immunomodulation, immunotherapy, oncology

## Abstract

Drug repurposing is a significant strategy in drug discovery, as it saves substantial amounts of time and money. Drugs already approved for other diseases can be repurposed to target cancer. Cancer remains one of the most fatal diseases, and it still does not have a cure. Developing new drug molecules and advancing them from preclinical to clinical stages can take many years, whereas drug repurposing offers a faster alternative. Many repurposed drugs have already progressed to clinical trials. This paper highlights recent advances in how FDA-approved drugs modulate the immune system to enhance host-based immune responses against cancer, and describes the mechanistic pathways through which these drugs act on immune cells. This paper also discusses how targeting macrophages, immune checkpoints on T-Cells, and other immune cell populations can strengthen immunotherapy. In addition, the paper reviews drugs that have advanced to clinical stages and are showing promising results across different cancers, as well as the challenges associated with clinical translation. This paper outlines how drug repurposing can influence the immune system within the context of cancer chemotherapy.

## Graphical Abstract

**Figure:**
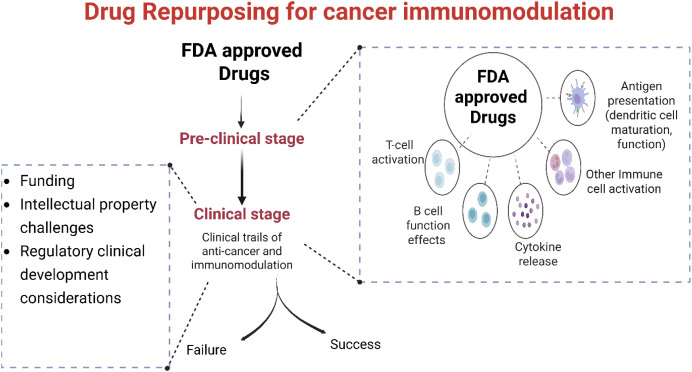
Drug repurposing for cancer immunomodulation. FDA-approved drugs screened for potential anticancer and immunomodulatory effects. In the preclinical stage, these compounds undergo *in vitro* testing to evaluate their impact on cancer cells and their modulation of the immune system, followed by *in vivo* studies in animal models to assess therapeutic efficacy and immune system engagement. The most promising candidates advance to clinical trials, where their ability to modulate innate and adaptive immune responses, including antigen presentation, immune cell activation, cytokine release, and T-Cell and B-cell function, is evaluated for potential clinical benefit in cancer treatment. The clinical stage depends on funding, intellectual property challenges, and regulatory clinical development considerations. All the stages ultimately may lead to success or failure in the drug repurposing.

## 1 Introduction

The development and successful clinical use of immune checkpoint inhibitors targeting CTLA-4, PD-1, and PD-L1 have transformed cancer therapy and were recognized through the 2018 Nobel Prize in Physiology or Medicine (1, 2). In recent years, the introduction of immunotherapy, especially immune checkpoint inhibitors targeting PD-1/PD-L1, has offered a promising avenue for treating advanced cancers. These PD-1/PD-L1 inhibitors primarily work by blocking the PD-1/PD-L1 signaling pathway, thereby relieving T-cell suppression on T cells and boosting their ability to fight tumors (3–8). Moreover, substances such as maprotiline boost the antitumor immune response in mouse melanoma by reducing PD-L1 expression. This research could uncover a new PD-L1 inhibitor, potentially offering an innovative treatment option for tumors in clinical settings (9). Although recent years have witnessed remarkable and highly successful immunotherapy trials, a substantial number of cancer patients either fail to respond or experience only temporary benefits, with their disease eventually returning because of rapidly emerging resistance. This resistance, whether present from the outset or acquired over time, remains poorly understood and cannot be effectively addressed without further in-depth investigation (10). Immunotherapy resistance is generally divided into primary and acquired forms. Primary, or intrinsic, resistance refers to cases in which a malignant tumor fails to respond to immunotherapy from the outset (11, 12). Resistance driven by multiple underlying mechanisms poses a major challenge to the success of immunotherapy in cancer treatment. This difficulty highlights the need for new therapeutic strategies, and one promising approach is drug repurposing.

In the highly competitive therapeutic and pharmaceutical landscape, repurposing existing drugs for immunotherapy is often preferred over developing entirely new compounds. Drug repurposing involves identifying new therapeutic uses for already approved, well-characterized medications that have been evaluated across various medical, experimental, or clinical settings (13). Whereas developing a new drug from scratch usually requires 6 to 9 years and costs $ 2–3 billion, drug repurposing allows researchers to move directly into preclinical evaluation and clinical trials. This approach significantly reduces time, expense, and potential adverse effects, and it often results in a higher likelihood of clinical success because the compounds have already been assessed for safety and pharmacokinetic profiles in humans (14, 15). Reusing existing drugs, reviving discontinued compounds, and extending patent longevity make drug repositioning an appealing strategy for drug development. In recent years, nearly 30% of new approvals by the USFDA have resulted from drug repurposing efforts. The growing interest in this area has also driven the development of advanced computational approaches designed to identify novel therapeutic applications for established drugs (15–17). Research suggests that drug repurposing could alter immune cell functions or modify the tumor microenvironment, thereby facilitating the overcoming of resistance to immune checkpoint inhibitors. This strategy provides a faster and more economical route for identifying therapeutic candidates that can be rapidly advanced into clinical testing. To enhance the success of tumor immunotherapy, developing systematic approaches for identifying repurposable drug candidates is essential (18, 19).

Recent studies have reshaped the understanding of how certain forms of cancer cell death influence the immune system, leading to the identification of a distinct subtype of apoptosis, immunogenic apoptosis (20). This type of cell death, triggered by particular chemotherapeutic agents, can generate a vaccine-like effect *in vivo* by activating a robust antitumor immune response (20). Recent advances in cancer drug discovery promise to establish a new cornerstone of cancer therapy. Repurposing drugs originally developed for modulating nerve signal transduction represents a promising approach to target oncogenic signaling pathways in cancer treatment (21). This paper emphasizes older drugs that can be repurposed for cancer therapy by modulating key immune cell populations, reprogramming tumor-associated macrophages from the M2 to the M1 phenotype, or shifting Tregs toward Th1, Th17, or CD8^+^ T-cell responses. These immunomodulatory effects could influence cancer-related pathways, thereby improving therapeutic outcomes. This review highlights the relevant pathways and drug candidates that may serve as immune modulators within a drug repurposing strategy or in combination therapies to against cancer. To understand these immunomodulatory concepts, it is essential to grasp the molecular mechanisms that govern immune cell plasticity in the tumor microenvironment with reference to drug repurposing. Mechanistic insights into immune cell activation may provide a biological foundation for rational drug repurposing.

## 2 Mechanistic insights into immunomodulatory drug repurposing

Understanding the molecular mechanisms underlying immune modulation is essential for the repurposing of existing FDA-approved drugs. In this section, the focus will be on discussing how immune cell populations within the tumor microenvironment could be therapeutically reprogrammed. Particular focus is on macrophage plasticity, T-cell functional polarization, and the signaling and metabolic pathways that govern these processes. These mechanisms may provide the basis for understanding to identify important targets and optimize repurposed drug strategies in cancer immunology.

### 2.1 Reprogramming macrophage polarization in cancer

Among immune populations, tumor-associated macrophages are a major player in tumor progression and therapeutic resistance (22). Therefore, understanding the mechanisms that control macrophage polarization between pro-inflammatory M1 and immunosuppressive M2 phenotypes is critical for developing effective immunomodulatory repurposing strategies (23). Macrophage reprogramming within the tumour microenvironment is determined by intricate mechanisms that shape their phenotype and function, including M1 and M2 polarisation, metabolic and epigenetic shifts, and diverse signalling networks (24). Tumour-associated macrophages orchestrate tumour progression, metastasis, and therapy resistance, positioning them as central targets for innovative combination treatment strategies (24, 25). Drugs such as Perhexiline, Nitazoxanide, and Telaglenastat disrupted IL-4 induced anti-inflammatory macrophage polarization by reducing CD163 and CD209 expression and CCL17 secretion. Importantly, Telaglenastat also enhanced macrophage-mediated tumor-killing activity, similar to that observed with LPS or IFN-γ activated macrophages (26). Similarly, methimazole markedly enhances coronary collateral circulation after myocardial infarction by promoting a shift from M1-like to M2-like macrophage polarization and elevating vascular endothelial growth factor A secretion. Mechanistically, methimazole targets MAPK 1 to suppress the MAPK1 ROS axis and inhibit ferroptosis, an effect reversed by the MAPK activator honokiol, underscoring the central role of this pathway (27). The tumour microenvironment metabolically reshapes TAMs, and targeting essential pathways related to glucose, lipid, or amino acid metabolism can redirect them toward an antitumour phenotype, thereby strengthening the overall immune response against cancer (28, 29). Resveratrol regulates macrophage polarization through multiple pathways, promoting M1 or inhibiting M2 phenotypes via SASP, STAT3, and S1P–YAP inhibition, thereby suppressing tumor growth. At the same time, through NF-κB inhibition or PI3K, Akt, and AMPK activation, it can drive M2 polarization to reduce inflammation (30). Resveratrol can enhance the senescence-associated secretory phenotype and block the STAT3 and sphingosine-1-phosphate YAP signalling pathways, which support M1 polarization or limit M2 polarization to suppress tumour growth. In contrast, by inhibiting the NF-κB pathway or activating the PI3K/Akt and AMP-activated protein kinase pathways, resveratrol can favour M2 polarization or reduce M1 polarization, thereby helping to resolve inflammatory responses (30). The low bioavailability of resveratrol is considered a significant barrier to its potential clinical application in cancer treatment (31). Similarly, Pseudolaric acid B and its modified derivatives were shown to strongly suppress ARG1 and enhance NOS2 expression in alternatively activated macrophages, indicating their capacity to shift tumour-associated macrophages toward an M1-like antitumour state. The hydrazineyl amide derivative emerged as the most effective compound, reversing M2-like macrophage markers, restoring CD8^+^ T-Cell proliferation and cytotoxicity, reshaping the tumor immune microenvironment, and significantly reducing tumor growth (32). Repurposing FDA-approved drugs that can shift tumour-associated macrophages toward an M1 phenotype and promote the release of TNF-α and IFN-γ, while enhancing the expression of costimulatory molecules, offers a powerful strategy to activate CD8^+^ T-Cells for anti-cancer responses. Such macrophage-centred approaches may represent a transformative approach in modern cancer chemotherapy.

Deciphering the pathways that regulate macrophage polarisation and reprogramming provides valuable opportunities to design therapies that can limit tumour progression. Advancing this knowledge toward clinical translation will be essential for developing more effective cancer treatments (24). In colorectal cancer (CC), evidence illustrates how tumor-intrinsic signaling mechanisms actively skew macrophages toward an immunosuppressive M2 phenotype that promotes therapeutic resistance (33, 34). For example, CC cells induce ADAR1-mediated RNA editing, driving macrophages into a drug-resistant M2 phenotype. In contrast, JAK inhibitor treatment reverses this shift, restoring oxaliplatin sensitivity (34). Hypoxic colorectal cancer cells upregulate SRC3 to drive M2 macrophage polarization and chemoresistance, whereas the natural drug bufalin lowers SRC3 and HIF1 alpha levels to block M2 polarization and restore chemotherapy sensitivity (35).

To reestablish anticancer immunity, a number of natural and repurposed drugs directly target important inflammatory signaling pathways inside TAMs, in addition to tumor-derived changes in macrophage polarization. Norcantharidin significantly suppresses colorectal cancer growth in both mouse and human tumour models while promoting strong M1 macrophage infiltration and polarization. Mechanistically, norcantharidin enhances CSF2 secretion and inhibits the JAK2-STAT3 pathway, thereby driving M1 polarization and reducing the proliferation, invasion, and migration of colorectal cancer cells (36). Metformin reduces colorectal tumorigenesis in an ETBF/AOM/DSS mouse model by alleviating colonic inflammation, repairing mucosal damage, and lowering tumor burden. It acts by suppressing the TLR4–MyD88–NFκB/MAPK pathway, inhibiting M2 macrophage polarization, and increasing protective short-chain fatty acids (37). Stachydrine inhibits colorectal cancer liver metastasis by blocking M2 macrophage polarization through the JAK2/STAT3 pathway, thereby reducing tumor migration, invasion, and angiogenesis. Its combination with anti-PD1 therapy further restores immune infiltration and enhances antitumor efficacy (38). In addition to pathway-level modulation, targeting transcriptional regulators and immune checkpoint–associated molecules further strengthens macrophage reprogramming strategies, such as Bufalin, which reverses immune escape in metastatic colorectal cancer by targeting SRC-3 to suppress KLF4 release, thereby inhibiting M2 macrophage polarization and PD-L1 upregulation in TAMs. This reprograms the tumor microenvironment, restores CTL activity, and enhances antitumor immunity (39). Tetrahydrocurcumin (THC) suppresses colorectal cancer by targeting the SPP1 CD4^+^ axis, inhibiting ERK signaling, and preventing M2 macrophage polarization. By disrupting this SPP1-driven immunosuppressive loop, THC restores anti-tumor immunity and reduces CRC progression (40). Similarly, drugs that enhance M1 polarization through alternative signaling mediators further remodel the tumor microenvironment. For example, Lobeline, a natural alkaloid, remodels the CRC tumor microenvironment by promoting M1 TAM polarization and suppressing M2 polarization through SLURP1 upregulation. By binding MAPK14 and enhancing SLURP1 secretion, lobeline reduces tumor growth and synergizes with anti-PD1 therapy for stronger antitumor effects (41). Cucurbitacin B suppresses colorectal cancer growth and metastasis by directly inhibiting JAK2/STAT3 signaling in tumor cells and blocking the polarization of M2 macrophages. By reducing M2-driven immunosuppression and enhancing CD4^+^ and CD8^+^ T-Cell responses, Cucurbitacin B exerts strong anti-tumor and anti-metastatic effects (42). Collectively, these findings underscore that targeting macrophage plasticity by modulating associated pathways is a promising immunomodulatory drug repurposing strategy in cancer.

Fenretinide (4-HPR) suppresses IL-4/IL-13-induced M2 macrophage polarization by inhibiting STAT6 phosphorylation, thereby reducing M2 marker expression and angiogenesis. In APCmin/+ mice, 4-HPR treatment decreased tumorigenesis and M2 macrophage infiltration, suggesting that inhibition of M2 polarization is a key mechanism underlying its chemopreventive effects in colon cancer (43). These findings further reinforce that pharmacologic inhibition of STAT-driven M2 polarization represents a recurring and targetable mechanism in colorectal cancer. The authors showed that EZH2 inhibitors suppress colorectal cancer not only by inhibiting tumour cell growth but also by reprogramming macrophages in the tumour microenvironment. EPZ6438 shifts macrophages from an M2 to an M1 phenotype by reducing H3K27me3 on STAT3 promoters, whereas GSK126 has the opposite effect, revealing distinct immunomodulatory roles for the two EZH2 inhibitors (44). Beyond epigenetic regulation, metabolic reprogramming has also emerged as a critical determinant of macrophage functional polarization. Clinically approved drugs targeting mitochondrial metabolism, including Perhexiline, VLX-600, CB-839, Trimetazidine, and HX531, can repolarize M2-like macrophages to an M1-like antitumor phenotype and inhibit M0-to-M2 polarization. This metabolic reprogramming enhances macrophage tumoricidal activity and shifts the immunosuppressive tumor microenvironment toward a pro-inflammatory state, supporting potential cancer immunotherapies (45). 

Colon cancer progression and therapy resistance are strongly influenced by the tumour microenvironment, particularly tumour-associated macrophages, whose plasticity enables immunosuppression, angiogenesis, metastasis, and drug tolerance. Growing therapeutic efforts now focus on depleting or reprogramming M2-like macrophages toward an M1 phenotype and blocking their key signaling pathways, offering promising avenues to overcome TAM-driven resistance and enhance immunotherapy in colon cancer (46). With the well-established safety profile and optimal dosing of these approved drugs, their combination with current cancer therapy is suggested to provide an economical, safe, and efficacious approach to overcome drug resistance and prolong patient survival (47). While these findings strongly support macrophage-targeted repurposing strategies in colorectal cancer, similar immunosuppressive mechanisms are also prominent in other highly lethal malignancies. Repurposing existing drugs offers a promising strategy in colorectal cancer by converting tumour-associated M2 macrophages back to an M1 antitumour phenotype, thereby strengthening host-driven immune therapies against cancer.

**Figure 1:**
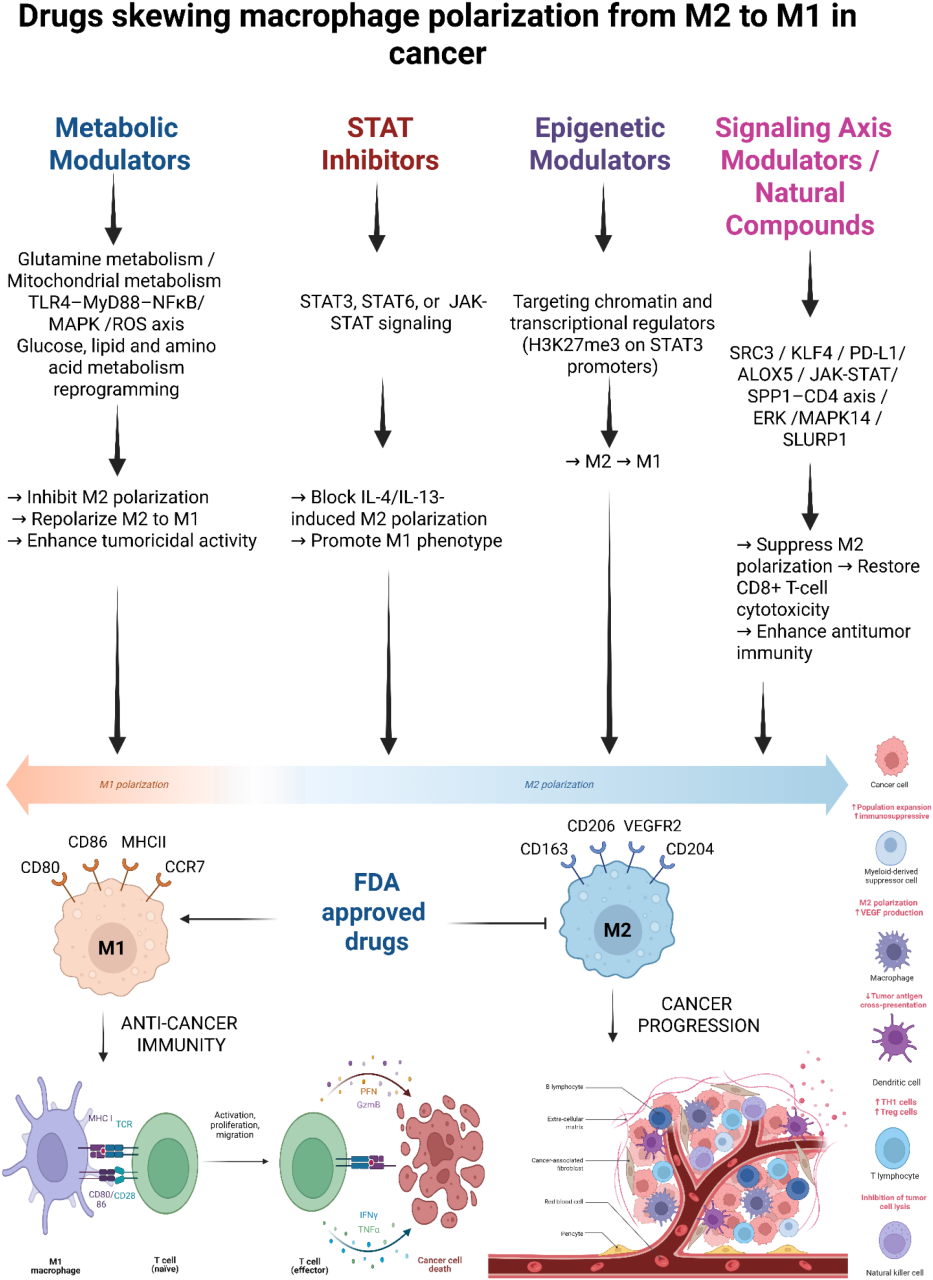
Repurposed and FDA-approved drugs reprogram tumor-associated macrophages (TAMs) to modulate macrophage polarization and reshape the tumor microenvironment. An overview of the major classes of drug modulators that influence macrophage polarization in the tumor microenvironment (TME). Metabolic Modulators (e.g., metformin, perhexiline, telaglenastat), STAT Inhibitors (e.g., resveratrol, cucurbitacin B, stattic), Epigenetic Modulators (e.g., EZH2 inhibitors, saikosaponin-D), and Signaling Axis Modulators/Natural Compounds (e.g., bufalin, ginsenosides, PI3K/Akt inhibitors). These drugs target key metabolic and signaling pathways (e.g., STAT3/STAT6, NF-κB, PI3K/Akt/mTOR, MAPK, ROS, EZH2-mediated chromatin remodeling), leading to functional reprogramming of TAMs along the M1–M2 polarization spectrum. The horizontal axis depicts the continuum from pro-inflammatory M1 polarization (anti-tumorigenic) to immunosuppressive M2 polarization (pro-tumorigenic). Repolarization toward the M1 phenotype enhances antigen presentation (e.g., MHC-II, CD80/CD86), promotes CD8^+^ T cell activation, and stimulates cytotoxic responses characterized by IFN-γ, TNF-α, perforin (PFN), and granzyme B (GzmB) release, ultimately resulting in tumor cell death. In contrast, M2 macrophages support tumor progression by promoting angiogenesis (e.g., VEGF production), suppressing immune responses, inhibiting T cells, and facilitating tumor invasion and metastasis.

In recent years, both the incidence and mortality of pancreatic cancer have continued to rise. The pronounced heterogeneity of this disease, along with its robust immune evasion and high metastatic potential, makes it one of the most lethal malignancies worldwide (48). Current chemotherapy approaches remain inadequate, and there is an urgent need for more effective and less toxic treatments to improve the poor therapeutic outcomes in pancreatic cancer. Repurposing existing non-oncology drugs offers a promising strategy, and several compounds are now being explored as potential candidates for use in pancreatic cancer therapy (49). Saikosaponin-D can overcome barriers by suppressing M2 macrophage polarization via inhibition of STAT6 and the PI3K-AKT-mTOR pathway. Using *in vivo* and *in vitro* models, the work demonstrates that saikosaponin D reduces tumor invasion, enhances immune activity, and may be a promising therapeutic candidate for pancreatic ductal adenocarcinoma (50). Additional evidence further supports the concept that disrupting macrophage-driven protumoral signaling pathways could significantly alter pancreatic tumor progression. Advances in immunotherapy, drug repurposing, and innovative delivery systems are transforming the ability to strengthen anticancer immune responses (51). A study reveals that high ALOX5 expression in pancreatic cancer, predominantly within macrophages, drives M2 polarization through the JAK-STAT pathway and promotes tumor invasion and metastasis. Zileuton counters these effects by inhibiting ALOX5, thereby reducing macrophage M2 activity, limiting cancer spread, and improving survival, underscoring its therapeutic potential (52). Ginsenoside Rh2, a bioactive compound from ginseng, has been shown to shift TAMs from the M2 to the M1 phenotype and to inhibit cancer cell migration by suppressing TAM activity in the tumor microenvironment (53). When combined, these results show that a shared treatment vulnerability in colorectal and pancreatic malignancies is macrophage reprogramming. Macrophage reprogramming within the tumour microenvironment is determined by intricate phenotypic and functional shifts shaped by M1 or M2 polarisation, metabolic and epigenetic changes, and multiple signalling pathways. Since TAMs strongly influence tumour growth, metastasis and treatment resistance, they represent a key focus for combination therapeutic strategies (**Table 1**) (24). Utilizing existing drugs to modulate macrophage polarization in pancreatic ductal adenocarcinoma holds strong therapeutic promise by shifting the tumor microenvironment from immunosuppressive to immunoactive, thereby supporting the effective elimination of pancreatic cancer (**Figure 1**). While macrophage reprogramming primarily targets the innate immune compartment of the tumor microenvironment, antitumor immunity ultimately depends on activating adaptive immune responses, such as restoring cytotoxic T-cell activity and overcoming regulatory T-Cell mediated suppression (54) (48). Therefore, drugs that enhance adaptive immune effector functions represent a complementary and synergistic extension of macrophage-targeted interventions.

**Table 1:** Repurposed drugs and effects on macrophage polarization (M1/M2) and tumor microenvironment.

Drug	Effect on macrophages and TME	Mechanism	Reference
Perhexiline, Nitazoxanide, Telaglenastat	Block IL-4 induced M2 polarization; ↓CD163, ↓CD209, ↓CCL17; Telaglenastat ↑ macrophage tumor killing	Metabolic interference	(26)
Methimazole	Shifts M1 to M2 after myocardial infarction; ↑VEGF A	Targets MAPK1; suppresses MAPK1 ROS; inhibits ferroptosis	(27)
JAK inhibitors	ADAR1 in CRC drives M2 polarization, drug resistance	Reversed by JAK inhibitors	(34)
Bufalin	SRC3 in CRC promotes M2 and chemoresistance	Bufalin reduces SRC3 and HIF1α	(35)
Norcantharidin	Promotes strong M1 infiltration; inhibits CRC growth	↑CSF2, ↓JAK2 STAT3	(36)
Metformin	Reduces CRC tumorigenesis; inhibits M2	Suppresses TLR4–MyD88–NFκB/MAPK; ↑SCFA	(37)
Stachydrine	Blocks CRC liver metastasis	Inhibits JAK2/STAT3 mediated M2	(38)
Bufalin	Reverses immune escape; inhibits M2; ↓PD L1 in TAMs	Targets SRC3 KLF4	(39)
Tetrahydrocurcumin	Suppresses CRC; blocks M2	Targets SPP1 CD4 + 4; ↓ERK	(40)
Resveratrol	Regulates M1/M2 balance	SASP; ↓STAT3; ↓S1P–YAP; NFκB inhibition; PI3K/Akt/AMPK activation	(30)
Pseudolaric acid B	Converts M2 to M1; ↑CD8^+^ proliferation	↓ARG1; ↑NOS2; hydrazineyl amide strongest effect	(32)
Lobeline	Promotes M1; inhibits M2	↑SLURP1; binds MAPK14; synergizes with anti PD1	(41)
Cucurbitacin B	Inhibits M2; suppresses growth and metastasis	Blocks JAK2/STAT3	(42)
Fenretinide (4 HPR)	Inhibits M2; ↓angiogenesis	Blocks STAT6; reduces colon tumorigenesis	(43)
EZH2 inhibitors	EPZ6438 shifts M2 to M1; GSK126 opposite effect	Via H3K27me3 effects on STAT3 promoter	(44)
Mitochondrial metabolism inhibitors	Repolarize M2 to M1	Perhexiline, CB839, VLX 600, TMZ, HX531	(45)

IL-4, interleukin 4; M1, classically activated macrophage (pro-inflammatory); M2, alternatively activated macrophage (anti-inflammatory/tumor-promoting); CD163, cluster of differentiation 163; CD209, cluster of differentiation 209; CCL17, C-C motif chemokine ligand 17; VEGF A, vascular endothelial growth factor A; ADAR1, adenosine deaminase acting on RNA 1; CRC, colorectal cancer; SRC3, steroid receptor coactivator 3; TAMs, tumor-associated macrophages; PD-L1, programmed death-ligand 1; CD8^+^, cluster of differentiation 8 positive; 4-HPR, 4-hydroxyphenyl retinamide (fenretinide); EZH2, enhancer of zeste homolog 2; EPZ6438, tazemetostat (EZH2 inhibitor); GSK126, EZH2 inhibitor; TME, tumor microenvironment.

### 2.2 Enhancing adaptive immune responses

While reprogramming tumour-associated macrophages can relieve immunosuppression at the innate level, anticancer immunity also requires effective adaptive responses mediated by T lymphocytes. T cells exert antigen-specific cytotoxicity but are frequently rendered dysfunctional by chronic stimulation and inhibitory cues in the tumour microenvironment, a phenomenon known as T cell exhaustion or anergy that limits antitumour activity (55). At the same time, regulatory T cells accumulate in tumours and suppress effector T-cell functions through cytokines like IL-10 and TGF-β and competition for IL-2, thereby contributing to immune escape (56).

This section highlights repurposed drugs and small molecules that modulate T-cell function, immune checkpoints, or tumor immunogenicity, illustrating how mechanistic insights from both mouse models and clinical trials can inform combination therapeutic strategies. Tumors remain unresponsive, underscoring the need for additional strategies to strengthen antitumor immunity. The key requirements for an effective T-Cell response via FDA-approved drug repurposing that may modulate immune function. Drugs that could be combined with checkpoint inhibitors to improve patient outcomes, drawing on available clinical evidence and supporting mechanistic findings from mouse models (57). Among these candidates, repurposed drugs such as naltrexone and disulfiram have shown promising immunomodulatory effects. For example, naltrexone, an opioid receptor antagonist widely used to treat alcohol dependence, has recently gained attention for its potential anticancer effects. Low-dose naltrexone has been shown to suppress cancer cell proliferation, reduce tumor growth by modulating key signaling pathways, and influence immune responses (58, 59). Similarly, a clinical study in metastatic ER-positive breast cancer reported that LDN was well-tolerated and produced modest therapeutic benefits (58, 60). In addition, Murugan et al. further demonstrated that combining naltrexone with the β2 adrenergic receptor blocker propranolol markedly inhibited breast cancer progression by downregulating EMT-related proteins, decreasing inflammatory cytokines, and increasing apoptotic markers, resulting in strong antitumor activity in preclinical models (58, 61). Collectively, these findings indicate that disulfiram and naltrexone may help resensitize drug-resistant breast cancers.

Beyond T-cell activation, modulation of antigen-presenting cells can further enhance adaptive immunity. Valproic acid (VPA) affects dendritic cell differentiation and antigen presentation (62). The application of valproic acid (VPA) in cancer therapy warrants careful evaluation, as multiple studies have demonstrated its anticancer potential across various tumor types. Current evidence suggests that VPA may not only reduce tumor cell viability but also influence the behavior and function of immune cells (16, 63). Investigations showed that VPA interferes with the development and maturation of dendritic cells (DCs). In C57BL/6 mice, VPA impairs the differentiation of bone marrow multipotent progenitors into both plasmacytoid and myeloid DCs by downregulating the transcription factors PU.1 and IRF8. VPA also decreases the surface expression of CD86 and MHCI (63).

While antigen presentation sets the stage for adaptive immunity, overcoming inhibitory checkpoint pathways is critical for restoring T-cell function. PD-1/PD-L1 antibodies and small-molecule inhibitors provide complementary strategies to reverse T-cell exhaustion (64). Antibodies that block PD-1 or its ligand PD-L1 can restore exhausted T-Cells and reinvigorate antitumor immunity. Owing to their remarkable clinical success, ten PD-1 inhibitors (nivolumab, pembrolizumab, cemiplimab, sintilimab, camrelizumab, toripalimab, tislelizumab, zimberelimab, prolgolimab, and dostarlimab) and three PD-L1 inhibitors (atezolizumab, durvalumab, and avelumab) have been approved for the treatment of multiple cancer types (65). The PD-1 immune checkpoint pathway is a promising therapeutic target in NSCLC, as it limits T-Cell activation by dampening immune responses, promoting self-tolerance, and preventing autoimmunity. However, this pathway is also linked to important inflammatory effects and has been explored in other inflammation-driven conditions, such as autoimmune diseases, chronic infections, and sepsis (66, 67). Nivolumab, a human IgG4 antibody, has shown superior overall survival in patients with advanced squamous NSCLC compared to docetaxel (66, 68). In cancer immunotherapy, combining inhibitors of pro-inflammatory cytokines, including TNF-α, TGF-β, and CSF, with anti-PD1 or anti-PDL1 agents has demonstrated enhanced therapeutic outcomes compared to monotherapy, as these cytokines can amplify immune responses while reducing immunosuppressive activity. Ongoing clinical trials are further evaluating the efficacy of cytokine combinations with various PD-1/PD PD-L1 inhibitors (66, 69, 69). Therapies designed to modulate or enhance anticancer immunity have made remarkable progress in recent decades, exemplified by the success of anti-PD1 and anti-PD-L1 monoclonal antibodies (51).

**Figure 2:**
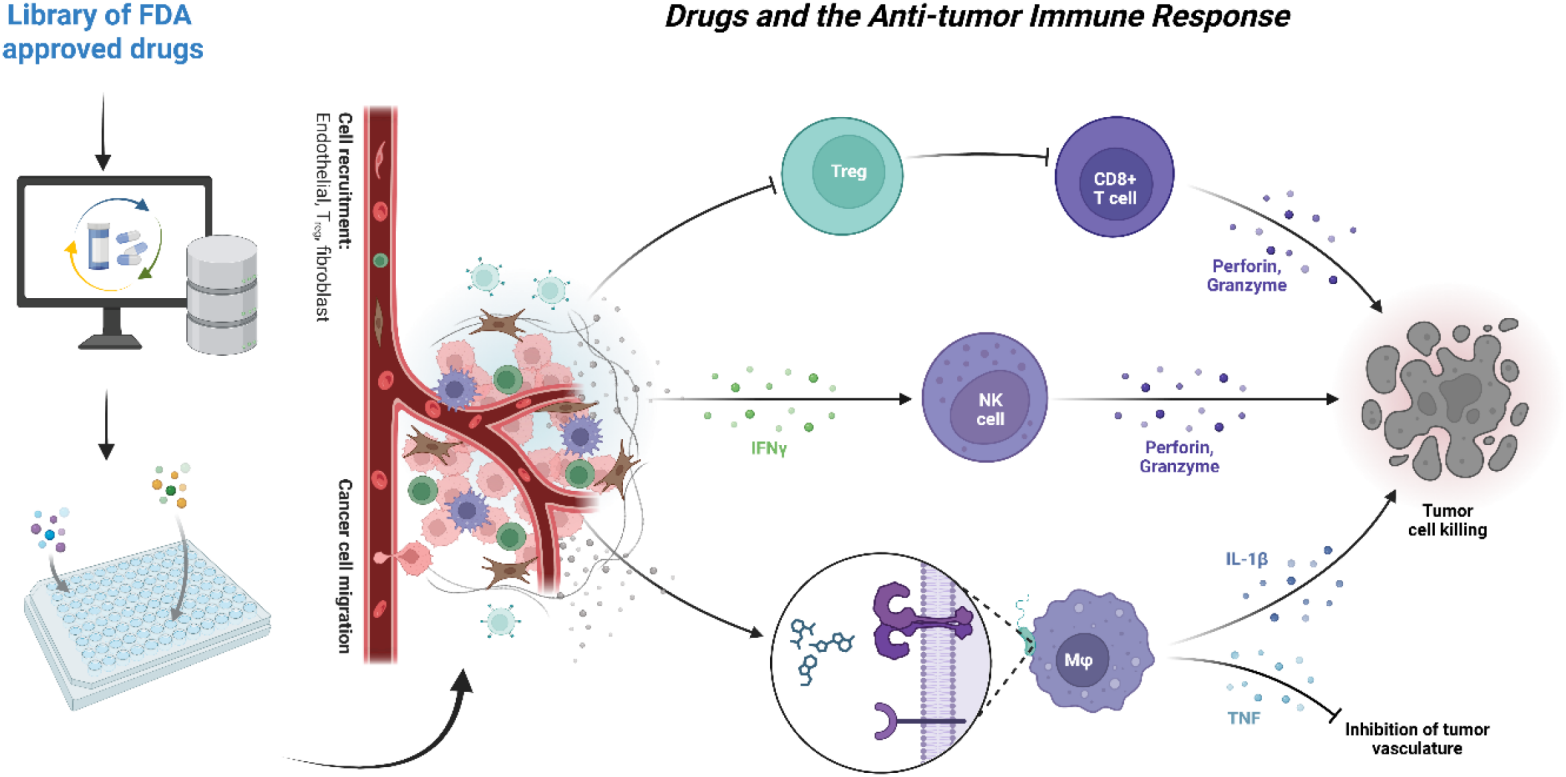
Drugs and the anti-tumor immune response within the framework of drug repurposing. FDA-approved drugs are screened computationally and experimentally to identify candidates with potential immunomodulatory activity. Selected compounds undergo *in vitro* testing to examine their effects on cancer cells and immune cell interactions. These drugs can influence the tumor microenvironment by modulating cell recruitment and migration, altering endothelial and stromal cell behavior, and engaging key immune populations. Repurposed agents may enhance CD8^+^ T-cell and natural killer cell-mediated cytotoxicity by increasing perforin and granzyme release, promote the production of macrophage-derived cytokines such as IL-1β and TNF, and inhibit tumor vasculature, thereby regulating T regulatory cell function. Together, these immune-mediated mechanisms contribute to improved tumor cell killing and overall antitumor responses.

Recent in silico and preclinical studies have identified additional repurposed drugs that modulate PD-L1 expression and stability, offering potential alternatives to antibody-based therapy (70). Liothyronine was recently highlighted as a potential PD-L1 binding agent through an in-silico drug screening approach. The analysis indicated that T3 may form a stable complex with PD-L1, primarily through π-π stacking between its central phenyl ring and the tyrosine 123 residue of PD-L1, along with additional hydrogen bonding and hydrophobic contacts that reinforce the interaction (70, 71). Tyrosine Y123 is a key residue required for PD-L1 dimerization and for PD-1/PD-L1 binding (70, 72). Based on these findings, liothyronine has been proposed not only to mitigate hypothyroidism risk but also to inhibit PD-1 1/PD PD-L1 engagement and decrease levels of its precursor hormone T4, thereby potentially enhancing antitumor immunity (70, 71). However, this computational prediction still lacks experimental confirmation. Blocking the PD-1/PD-L1 pathway with antibodies has produced durable antitumor effects; however, issues such as poor permeability, immune toxicity, complex production, and high cost limit their widespread use. Similarly, the FDA-approved drug Ponatinib is a potent small-molecule PD-L1 inhibitor that binds strongly to PD-L1 and, *in vivo*, delays tumor growth more effectively than an anti-PD-L1 antibody, while enhancing T-Cell activity, offering a promising alternative to antibody-based therapy (73). Cancer cell PD L1 expression drives T-Cell exhaustion, making its regulatory mechanisms attractive therapeutic targets. Through a drug repurposing screen, amlodipine was identified as a strong suppressor of PD-L1, acting by blocking calcium-driven, calpain-mediated Beclin 1 cleavage that normally prevents autophagic degradation of PD-L1 on recycling endosomes. Inhibiting calcium influx with amlodipine reduced PD-L1 levels, enhanced CD8^+^ T-Cell infiltration, and produced dose-dependent tumor suppression *in vivo*, effects that were reversed by restoring PD-L1 expression (74). These findings reveal a calcium-dependent pathway that regulates PD-L1 stability, suggesting that inhibiting calcium flux may be a promising immunotherapy strategy. A range of cancer-targeting channel blockers, such as amlodipine, may have potential as PD-L1 inhibitors. Many of these compounds still require experimental validation, but they could help shift exhausted T-Cells toward a CD8^+^ reactive phenotype, thereby enhancing antitumor immunity and slowing cancer progression (**Figure 2**). FDA-approved drugs demonstrate potential immunomodulatory activity and influence the tumor microenvironment by modulating cell recruitment and migration, altering endothelial and stromal cell behavior, and engaging key immune populations (75, 76). They may enhance CD8^+^ T-cell and natural killer cell-mediated cytotoxicity by increasing perforin and granzyme release, promote the production of macrophage-derived cytokines such as IL-1β and TNF, and improve tumor cell killing.

Despite considerable progress in improving cancer survival, the global burden remains high and continues to grow. Immune checkpoint inhibitors targeting PD-1/PD-L1 have transformed cancer therapy, but issues like poor oral availability, side effects, and high cost make small-molecule alternatives appealing; using QSAR models and virtual screening of FDA-approved drugs, we identified and validated small-molecule candidates such as sonidegib that modulate PD 1/PD L1, showing promise for accelerating drug discovery toward more accessible checkpoint inhibitors (77). Several repurposed drugs have shown the ability to modulate the tumor microenvironment, boost T-Cell responses, and overcome resistance to immune checkpoint inhibitors. For example, mechanistically, these agents act via distinct pathways: RANKL inhibitors can strengthen antitumor immunity, TGF-β inhibitors promote T-cell infiltration, and metformin enhances the activity of cytotoxic T lymphocytes (78). Complementary immunomodulatory approaches, including GM-CSF and thymosin α1, support antitumor responses by stimulating T-Cell function and promoting dendritic cell maturation. Aspirin may further improve the outcome of checkpoint blockade therapy by counteracting tumor-associated immunosuppressive pathways. Despite encouraging preclinical findings and ongoing clinical investigations, challenges persist in optimizing dosing regimens, minimizing unintended effects, and navigating regulatory complexities (57, 78). Despite these promising strategies and encouraging preclinical and clinical data, challenges remain in optimizing dosing, minimizing off-target effects, and addressing regulatory hurdles (79–81). Collectively, these repurposed drugs illustrate how modulation of T-cell function, antigen presentation, and checkpoint pathways may synergize with innate immunity-targeted therapies (macrophage reprogramming), providing a promising approach to enhance antitumor immunity.

## 3 Nanoformulation-based strategies utilizing drug repurposing

Drug repurposing combined with nanoformulation offers an innovative strategy to improve solubility, bioavailability, and tumor-targeted delivery, thereby enhancing the anticancer efficacy of existing drugs (82). Benproperine phosphate (BPP), a cough suppressant, exhibits potent anticancer activity in pancreatic cancer by inducing autophagy-mediated cell death. Mechanistically, BPP activates AMPK/mTOR-driven autophagy while disrupting RAB11A-dependent autophagosome-lysosome fusion, causing lethal autophagy arrest and growth inhibition in pancreatic cancer cells (83). To further improve delivery and therapeutic outcomes, a hyaluronic acid–modified ZIF-8 nanoplatform co-delivering BPP and gemcitabine (HA/ZIF-8@BPP/Gem) has been developed, which converts protective autophagy into lethal autophagy while activating T-cell–mediated immune responses in pancreatic cancer. This nano-enabled drug repurposing approach demonstrates synergistic antitumor efficacy with minimal toxicity in cell lines and preclinical tumor models (84).

Beyond direct tumor cytotoxicity, nanoformulations can also enhance antitumor immunity by improving T-Cell infiltration and function within the immunosuppressive tumor microenvironment (85). Intratumoral CD8^+^ T-Cells are crucial for the success of cancer immunotherapy; however, their function and infiltration are often suppressed by the immunosuppressive tumor microenvironment. Using drug repurposing, self-degradable PMI nanogels delivering imiquimod and metformin were developed to remodel the TME by activating dendritic cells, repolarizing M2 macrophages, and reducing PD-L1, ultimately enhancing CD8^+^ T-Cell activation and boosting the efficacy of anti-PD1 therapy (86).

In addition to immune-targeted nanoformulations, repurposed anti-inflammatory drugs can be reformulated at the nanoscale to enhance cytotoxicity and modulate the tumor microenvironment (87, 88). Screening NSAIDs for anticancer properties has emerged as a promising approach to discover new cancer therapies. Several widely used anti-inflammatory drugs, including aspirin, ibuprofen, diclofenac, and celecoxib, have demonstrated antitumor activity, with some currently undergoing clinical trials (89). Advances in cancer biology and molecular targeting have enabled the development of novel therapies, with drug repurposing offering a lower cost and improved safety. COX-2 inhibitors, particularly celecoxib, show promise as anticancer agents, and nanotechnology-based delivery strategies further enhance their chemopreventive and combinatorial therapeutic potential (90). Nanoformulation strategies that improve solubility and bioavailability can convert repurposed drugs into potent anticancer agents with reduced systemic toxicity (91). For example, nanoformulated ibuprofen, ketoprofen, and naproxen particles (<30nm) showed markedly improved solubility and drug release, with naproxen-PVP exhibiting over twice the anti-leukemia activity of doxorubicin. The poor water solubility of certain NSAIDs limits their application for cancer treatment. Reducing the particle size of such drugs to the nanoscale can improve solubility, thereby enhancing their anticancer activity. Additionally, improving solubility and bioavailability can help minimize the high dosages and associated side effects commonly observed with NSAID administration (92). A novel evaporation-assisted solvent antisolvent interaction method enabled the nanoformulation of ibuprofen, ketoprofen, and naproxen into stable particles with diameters of less than 30 nm, resulting in markedly improved solubility and drug release. These nanoformulated NSAIDs showed enhanced anticancer activity across multiple cancer cell lines, with the naproxen PVP formulation displaying over twice the anti-leukemia efficacy of doxorubicin (92). Similarly, Maria Mendes and colleagues proposed a dual strategy for glioblastoma by repurposing celecoxib and delivering it through ultra-small nanostructured lipid carriers, engineered to enhance solubility, thermo-responsive release, and tumor targeting. Their optimized usNLCs exhibited a stable nanoscale size, controlled heat-triggered drug release, and strong cytotoxicity in glioma cells, highlighting their potential to enhance GB therapy (93).

Additionally, a mesoporous silica nanoparticle-based system (MNP-Asp-PD-PG-F) was developed to deliver aspirin specifically to breast cancer cells, thereby enhancing targeted therapy via folic acid conjugation. This delivery system exhibited higher cytotoxicity and antiproliferative effects compared to free aspirin, demonstrating potential for effective, tumor-specific treatment of breast cancer (94). Similarly, Shevanuja Theivendran and colleagues developed a glutathione-responsive dendritic mesoporous organosilica nanoformulation loaded with pioglitazone to reprogram cancer-associated fibroblasts in the breast tumor microenvironment, thereby improving drug penetration. This strategy effectively downregulated CAF biomarkers and enhanced doxorubicin delivery, resulting in significant tumor growth inhibition (95). Beyond breast cancer, nanoformulations enhance the efficacy of repurposed drugs in other cancers. For example, Itraconazole, an antifungal drug with emerging anticancer potential, was successfully repurposed by formulating chitosan-coated PLGA nanoparticles, which improved its solubility, release profile, and cytotoxicity in H1299 lung cancer cells. These nanoparticles enhanced apoptosis, cell cycle arrest, and pro-apoptotic signaling compared to free itraconazole, indicating a promising strategy for lung cancer therapy (96). In addition, Gloria Saorin and colleagues report that formulating the novel PIN1 inhibitor VS10 into albumin-stabilized nanocrystals markedly enhances its antitumor efficacy in ovarian cancer models. When combined with pegylated liposomal doxorubicin, this nanoformulation produced strong synergistic tumor suppression, supporting its promise as an improved second-line therapy for high-grade serous ovarian cancer (97). In addition, combining the ultrasound-responsive nanosystems with drug repurposing offers a promising approach to enhancing immunotherapy. These nanocarriers demonstrate impressive effectiveness in delivering therapeutic agents directly to tumor locations through ultrasound guidance, which improves both the specificity and effectiveness of the treatment (98). Moreover, sorafenib, a first-line therapy for advanced hepatocellular carcinoma, shows limited clinical benefit; therefore, a Fe(III)-based metal-organic framework nanocarrier (Sor@Fe-MOF) has been developed to enhance its efficacy. Sor@Fe-MOF enhances ferroptosis and anti-tumor immunity, particularly by modulating GPX4, SLC7A11, ACSL4, and promoting CD8+ T cell infiltration, resulting in improved therapeutic outcomes (99). Therefore, such strategies may help improve the therapeutic outcomes of drugs to boost immunotherapy.

Other nano-enabled strategies target key cancer vulnerabilities, such as mitochondrial dysfunction, which drives cancer progression, making these organelles prime targets for therapy. Recent advances in mitochondria-targeting nanoformulations, including liposomes, dendrimers, polymeric, and inorganic nanoparticles, enable precise drug delivery and mitocan-based anticancer strategies; however, significant challenges remain for clinical translation (100). Amino bisphosphonates like zoledronate and alendronate possess antitumor and immune modulatory effects, but their clinical utility is limited by rapid renal clearance and bone sequestration. Liposomal reformulation overcomes these barriers, enhancing their anticancer efficacy, with pegylated liposomal alendronate emerging as the most promising candidate for clinical translation (101). Fluvastatin, a standard antihypercholesterolemia drug with known antitumor activity, shows significantly enhanced cytotoxicity against breast cancer stem cells when encapsulated in hyaluronan-conjugated liposomes. The hyaluronan-conjugated FLUVA-encapsulating nanoliposomes, especially in combination with doxorubicin, improved survival *in vivo* with minimal toxicity, supporting their potential as an effective breast cancer therapy (102). Together, studies discussed highlight how nanoformulation strategies may enhance the efficacy, bioavailability, and tumor-targeting of repurposed drugs, while simultaneously modulating the tumor microenvironment and antitumor immunity.

## 4 Advances in drug repurposing

Building on mechanistic understanding and advanced delivery strategies, drug repurposing offers a rapid and cost-efficient approach to enhance cancer immunotherapy, particularly by overcoming resistance to immune checkpoint inhibitors through modulation of immune activity and the tumor microenvironment (103, 104). Drug repurposing has emerged as a rapid and cost-efficient strategy to enhance cancer immunotherapy, particularly by overcoming resistance to immune checkpoint inhibitors through modulation of immune activity and the tumor microenvironment (105, 106). Systematic approaches, including computational screening and specialized databases, are accelerating the identification of repurposed antidiabetic, antihypertensive, antifungal, antiviral, and other drugs with the potential to expand current immunotherapy options (18). Advances in personalized medicine and artificial intelligence are expected to further strengthen drug repurposing efforts in oncology. Achieving full clinical impact will require coordinated efforts among researchers, clinicians, industry, and regulatory agencies (78).

Cancer’s complexity, heterogeneity, and therapy resistance make effective treatment challenging, despite numerous available strategies. Recent advances favor a shift from gene-centric “silver bullet” therapies to systems-level, pathway-based “shrapnel” approaches, supported by computational oncology and multidisciplinary collaborations, to develop more effective, personalized treatments (107). Advances in cancer diagnostics, genomics, and tumor biology have increased the need for personalized therapies. Since developing new cancer drugs is costly and time-intensive, drug repurposing offers a faster and more efficient path to identify promising treatment options (108).

Combining repurposed drugs, guided by multifactorial data analysis and artificial intelligence, can enhance therapeutic efficacy while expanding treatment options for genetically heterogeneous tumors (109). Artificial intelligence, particularly deep learning, offers powerful tools for innovative drug discovery and design, including virtual screening, *de novo* design, property prediction, and drug repurposing (110). A quantitative high-throughput screening assay was optimized to identify drugs that regulate exosome biogenesis or release in aggressive prostate cancer cells expressing CD63-GFP. Screening 4,580 compounds from LOPAC and NPC libraries revealed 22 agents capable of significantly increasing or decreasing intracellular GFP signal (111). Validation using TRPS analysis, flow cytometry, and immunoblotting confirmed the inhibitory effects of several compounds (tipifarnib, neticonazole, climbazole, ketoconazole, triadimenol) and activatory effects of others (sitafloxacin, forskolin, SB218795, fenoterol, nitrefazole, pentetrazol) on exosome production or secretion. These findings highlight drug repurposing as a promising strategy for targeting exosome pathways in the treatment of advanced cancer (111). Utilizing the drug repositioning workflow and a validated 3D QSAR pharmacophore model (Hypo 1) to screen DrugBank compounds, ultimately identifying Daclatasvir as a potent PD-L1 small molecule inhibitor. Daclatasvir bound to PD-L1 with high affinity, activated T-Cell-mediated killing in co-culture, suppressed tumor growth *in vivo*, and showed even stronger antitumor effects when combined with Lenvatinib, highlighting its promise as a repurposed PD-L1 inhibitor (112).

Repurposing refers to using an already approved drug for a different disease, a strategy driven by the high costs and long timelines associated with developing new medicines. Since new cancer drugs can take many years or even decades to clear clinical trials, repurposing offers a faster, safer alternative (113). With the rapid growth of oncological data like sequencing and proteomics, data-driven approaches are increasingly guiding drug repurposing efforts. This shift has led to the development of large-scale drug response resources, such as Profiling Relative Inhibition Simultaneously in Mixtures (PRISM) and Library of Integrated Network-Based Cellular Signatures (LINCS), enabling the direct and efficient discovery of repurposing opportunities (51). Immunotherapy has become a central cancer treatment strategy, with approved options including immune checkpoint inhibitors, cytokine therapies, and adoptive cell transfer such as CAR T-Cells. Yet, many patients exhibit limited or resistant responses, underscoring the need for more effective and rational immunotherapeutic approaches (51, 114–116).

Most clinical trials focused on evaluating safety, efficacy, and feasibility, with primary outcomes including overall survival, progression-free survival, and adverse drug reactions in adult participants. These studies were often limited by small sample sizes and heterogeneous populations. While overall survival generally did not improve with treatment, some trials showed better progression-free survival in the intervention group (117). Drug repurposing efforts in cancer have yielded promising preclinical results, and numerous candidates have now progressed to clinical trials across multiple cancer types (**Table 2**). With the numerous advantages of repurposing outlined in this paper, there is strong hope that these approaches will yield effective cancer therapies in the future.

**Table 2:** Clinical trials of drug repurposing in cancer.

Drug	Purpose and cancer type	NCT number and URL
Temozolomide	To evaluate the prognostic value of patient-derived organoids in predicting responses to conventional and repurposing drugs, including temozolomide.	Study Details NCT06782984 Drug Response Testing and Repurposing Using Glioblastoma Organoid ClinicalTrials.gov
Chlorpromazine	The study investigates whether adding chlorpromazine to the standard first-line treatment regimen improves outcomes in Glioblastoma Multiforme.	Study Details NCT04224441 Repurposing Chlorpromazine in the Treatment of Glioblastoma ClinicalTrials.gov
Metformin, Acetylsalicylic acid, Olaparib capsules, Letrozole	Women diagnosed with advanced stage IIIA to IV ovarian cancer, specifically high-grade serous carcinoma (HGSOC), who are undergoing diagnostic laparoscopy.	Study Details NCT03378297 IMPACT: A Randomized WOO Study of Novel Therapeutic Agents in Women Triaged to Primary Surgery for EOC ClinicalTrials.gov
Metformin	Evaluating metformin added to standard therapy in locally advanced or metastatic prostate cancer	Study Details NCT03137186 Repurposing Metformin as Anticancer Drug: in Advanced Prostate Cancer ClinicalTrials.gov
Metformin	Single-arm pilot study assessing the feasibility and safety of metformin in patients with clonal cytopenia of undetermined significance (CCUS) or lower-risk myelodysplastic syndromes (LR-MDS)	Study Details NCT04741945 Repurposing Metformin As a Leukemia-preventive Drug in CCUS and LR-MDS ClinicalTrials.gov
Fluoxetine	Phase I trial evaluating whether fluoxetine can alter tumor immune cells before surgery in colorectal cancer patients. The study investigates if fluoxetine modifies the tumor microenvironment and systemic immunity, potentially affecting tumor growth and spread.	Study Details NCT06225011 Fluoxetine for the Modification of Colorectal Tumor Immune Cells Before Surgery in Patients With Colorectal Cancer ClinicalTrials.gov
Metformin	Drug Repurposing for the Prevention of Chemotherapy-induced Peripheral Neuropathy (CIPN)	Study Details NCT04780854 Drug Repurposing for the Prevention of Chemotherapy-induced Peripheral Neuropathy (CIPN) ClinicalTrials.gov
Decitabine	Evaluating the therapeutic potential of decitabine repurposed for advanced, refractory pancreatic ductal adenocarcinoma (PDAC) that shows molecular signatures indicating KRAS dependency.	Study Details NCT05360264 tailOred dRug repurposIng of dEcitabine in KRAS-dependeNt refracTory pAncreaTic cancEr ClinicalTrials.gov
Chlorpromazine	Investigating chlorpromazine as an adjuvant therapy in patients with resected stage III colon cancer.	Study Details NCT05433402 Repurposing the Antipsychotic Drug Chlorpromazine as Adjuvant Therapeutic Agent for Resected Stage III Colon Cancer ClinicalTrials.gov
Letrozole, Bicalutamide, Everolimus,Itraconazole	Analysis of signal transduction pathway (STP) activity in ovarian cancer to guide phenotype-based targeted therapy decisions.	Study Details NCT03458221 Signal TrAnsduction Pathway Activity Analysis in OVarian cancER ClinicalTrials.gov
ATRA, gemcitabine nab-paclitaxel	Open-label, multi-center, randomized, stratified phase IIb trial evaluating ATRA combined with gemcitabine and nab-paclitaxel in patients with locally advanced pancreatic ductal adenocarcinoma (laPDAC).	Study Details NCT04241276 A Randomised Trial of ATRA in a Novel Drug Combination for Pancreatic Cancer ClinicalTrials.gov
Selpercatinib	Evaluating the impact of selpercatinib on cachexia and anorexia in patients with non-small cell lung cancer (NSCLC), colorectal cancer, or pancreatic cancer.	Study Details NCT07146893 Feasibility Study for Repurposing RET Inhibitors ClinicalTrials.gov
ATRA, gemcitabine nab-paclitaxel	Phase 1B trial repurposing ATRA as a stromal-targeting agent in combination with gemcitabine and nab-paclitaxel for pancreatic cancer (STAR_PAC).	Study Details NCT03307148 Stromal TARgeting for PAncreatic Cancer (STAR_PAC) ClinicalTrials.gov
Artesunate	Phase II randomized, double-blind, placebo-controlled trial evaluating artesunate as neoadjuvant therapy in patients with stage II/III colorectal cancer.	Study Details NCT07095309 Safety and Effectiveness Study of Pre-operative Artesunate in Stage II/III Colorectal Cancer ClinicalTrials.gov
Artesunate	Phase II single-arm, open-label study investigating artesunate for treating HPV-positive high-grade cervical intraepithelial neoplasia (CIN2/3).	Study Details NCT07095478 Safety and Efficacy of Oral Artesunate for Pre-cervical Cancer ClinicalTrials.gov
Ibrutinib + Indoximod metronomic cyclophosphamide, and etoposide	Phase 1B study repurposing ibrutinib in combination with indoximod, metronomic cyclophosphamide, and etoposide for pediatric patients with brain cancer.	Study Details NCT05106296 Chemo-immunotherapy Using Ibrutinib Plus Indoximod for Patients With Pediatric Brain Cancer ClinicalTrials.gov
Glutamate inhibitors	Phase Ib/II randomized, open-label trial repurposing glutamate signaling inhibitors in combination with chemoradiotherapy for patients with newly diagnosed glioblastoma.	Study Details NCT05664464 Glutamate Inhibitors in Glioblastoma ClinicalTrials.gov
Itraconazole (repurposed antifungal)	Investigating repurposing itraconazole as an adjuvant therapy to enhance treatment options in patients with acute myeloid leukemia (AML).	Study Details NCT07069933 Repurposing Itraconazole as an Adjuvant Therapy in Treatment of Patients With Acute Myeloid Leukemia: Is it a Hopeful Avenue for Enhancing Treatment Options? ClinicalTrials.gov
Tranexamic Acid	Evaluating perioperative tranexamic acid in melanoma patients to study the prognostic and therapeutic impact of modulating the plasminogen–plasmin pathway.	Study Details NCT05899465 Perioperative Treatment With Tranexamic Acid in Melanoma ClinicalTrials.gov
Nelfinavir (antiretroviral repurposed)	Phase I trial investigating nelfinavir in adults with solid tumors.	Study Details NCT01445106 A Phase I Trial of Nelfinavir (Viracept) in Adults With Solid Tumors ClinicalTrials.gov
DRUP Trial – multiple repurposed drugs	A Dutch national study conducted by the Center for Personalized Cancer Treatment (CPCT) to evaluate the potential efficacy of commercially available targeted anti-cancer drugs in patients with advanced cancers based on known molecular profiles.	Study Details NCT02925234 The Drug Rediscovery Protocol (DRUP Trial) ClinicalTrials.gov

NCT, National Clinical Trial number (ClinicalTrials.gov identifier); WOO, window of opportunity; CCUS, clonal cytopenia of undetermined significance; LR-MDS, low-risk myelodysplastic syndrome; KRAS, Kirsten rat sarcoma viral oncogene homolog; ATRA, all-trans retinoic acid; RET, rearranged during transfection; CIN 2/3, cervical intraepithelial neoplasia grades 2 and 3; CIPN, chemotherapy-induced peripheral neuropathy; TNF, tumor necrosis factor; DRUP, Drug Rediscovery Protocol trial.

## 5 Challenges in clinical translation and future directions

Drug repurposing is commonly viewed as an efficient and economical approach to discovering new therapeutic uses for existing medications. Nonetheless, academic investigators often underestimate the practical steps required to translate a repurposed drug into a clinically approved treatment for a different indication (118). This oversight may partly explain why drug repurposing has not consistently achieved its anticipated impact. Critical elements that are frequently neglected include funding and intellectual property challenges, regulatory and clinical development considerations, and the concept of clinical equipoise, which underpins the ethical conduct of randomized controlled trials (118, 119). Promising compounds are frequently set aside because they do not show adequate efficacy compared to existing treatments or have limited commercial appeal. Insufficient funding, restricted access to data, and difficulties in negotiating intellectual property rights are major barriers to drug repurposing realizing its full potential as a central drug development strategy (120).

According to ClinicalTrials.gov, the publicly accessible database of human clinical studies, there are currently 81 registered studies involving resveratrol. Most of these trials have primarily assessed its safety, tolerability, pharmacokinetics, and bioavailability, while only a few have specifically investigated its efficacy in treating specific types of cancer (31). Despite the benefits of drug repurposing, such as proven anticancer pharmacokinetic properties and acceptable safety and tolerability in preclinical or human studies, there remains, as with all drug development, a risk of failure in later-stage clinical trials, particularly due to competition from newly developed successful drugs (121).

Although substantial resources are invested in developing new therapies, only about 5 percent of cancer drugs that enter Phase I clinical trials ultimately receive approval for routine clinical use. At MD Anderson Cancer Center, early-phase clinical trials employing drug repurposing approaches have shown encouraging results in patients with both rare and common forms of treatment-resistant advanced cancers (122). For off-patent drugs, it is possible to secure a new method-of-use patent for a novel repurposed indication, provided that the proposed use is truly innovative and supported by sufficient evidence to establish credibility. However, enforcing such a patent can be problematic when the new indication relies on existing formulations of the generic drug. This difficulty arises because generic versions may already be widely marketed by other manufacturers and prescribed for non-patented indications (106).

A variety of computational resources have been developed to support the validation or refutation of drug repurposing hypotheses. Key datasets have been compiled to prioritize candidates for further development, including genome sequencing, transcriptional response profiles, functional pathway mappings, compound structures, target binding assays, cellular phenotypic profiles, and clinical outcomes such as adverse effects (123). The use of network pharmacology has transformed the field, reducing research and development timelines and significantly enhancing the efficiency of drug discovery (124). Drugs identified via computational approaches do not always show similar trends in the experimental setting; therefore, it’s very important to validate the computational findings in the experimental setting. Therefore, all challenges should be considered before pursuing drug repurposing for a new indication. In addition, research in drug discovery, especially in multi-omics, is advancing rapidly as researchers aim to decode the complexities of disease biology and improve treatment outcomes. Multi-omics strategies, which integrate information from genomics, transcriptomics, proteomics, metabolomics, and other omics disciplines, are vital for understanding the intricate molecular mechanisms underlying disease and aid in drug discovery (125). Artificial intelligence (AI) technologies have emerged as effective tools for navigating the complexities of cancer immunotherapy due to their robust data processing and pattern recognition capabilities. AI can assimilate and evaluate diverse, multifaceted sources of information, such as high-throughput sequencing, medical imaging, and clinical data, thereby providing essential support for precision oncology (126–129), which may help repurpose drugs to boost immunotherapy.

## 6 Summary

Drug repurposing offers a promising, cost and time efficient strategy for cancer therapy. While these agents have established safety profiles, thorough preclinical and clinical validation is essential to confirm their anticancer suitability. Given the central role of the immune system in controlling cancer, evaluating whether repurposed drugs could enhance host immunity through immunomodulatory effects is critical. Moving forward, integrating mechanistic insights, nanoformulations, and computational drug repurposing could increase the development of immune-enhancing cancer therapies. Future research should carefully consider regulatory, patent, immunotoxicology, and ethical issues, as well as optimal dosing, combination therapies, and funding constraints. Importantly, mechanistic studies should assess whether drug combinations can overcome tumor-induced immunosuppression and resistance, while nanoformulations may further improve targeted delivery, therapeutic index, and immune activation. Computationally predicted candidates should be rigorously validated at both preclinical and clinical stages, with AI and next-generation tools providing powerful support to accelerate drug repurposing and immunotherapy in the coming years. Coordinated efforts across regulatory, ethical, and translational domains might be essential to bring these strategies safely and effectively into clinical practice, ultimately enabling more personalized and potent immunotherapy approaches.
